# Fighting Pancreatic Cancer with a Vaccine-Based Winning Combination: Hope or Reality?

**DOI:** 10.3390/cells13181558

**Published:** 2024-09-16

**Authors:** Silvia Brugiapaglia, Ferdinando Spagnolo, Simona Intonti, Francesco Novelli, Claudia Curcio

**Affiliations:** 1 Department of Molecular Biotechnology and Health Sciences, University of Turin, Piazza Nizza 44bis, 10126 Turin, Italy; silvia.brugiapaglia@unito.it (S.B.); simona.intonti@unito.it (S.I.); franco.novelli@unito.it (F.N.); 2 School of Advanced Defence Studies, Defence Research & Analysis Institute, Piazza della Rovere 83, 00165 Rome, Italy; ferdinando.spagno57@edu.unito.it (F.S.)

**Keywords:** pancreatic ductal adenocarcinoma, vaccine, chemotherapy, radiotherapy, immune checkpoint inhibitors

## Abstract

Pancreatic adenocarcinoma (PDA) represents the fourth leading cause of cancer-related mortality in the USA. Only 20% of patients present surgically resectable and potentially curable tumors at diagnosis, while 80% are destined for poor survival and palliative chemotherapy. Accordingly, the advancement of innovative and effective therapeutic strategies represents a pivotal medical imperative. It has been demonstrated that targeting the immune system represents an effective approach against several solid tumors. The immunotherapy approach encompasses a range of strategies, including the administration of antibodies targeting checkpoint molecules (immune checkpoint inhibitors, ICIs) to disrupt tumor suppression mechanisms and active immunization approaches that aim to stimulate the host’s immune system. While vaccines have proved effective against infectious agents, vaccines for cancer remain an unfulfilled promise. Vaccine-based therapy targeting tumor antigens has the potential to be a highly effective strategy for initiating and maintaining T cell recognition, enhancing the immune response, and ultimately promoting cancer treatment success. In this review, we examined the most recent clinical trials that employed diverse vaccine types to stimulate PDA patients’ immune systems, either independently or in combination with chemotherapy, radiotherapy, ICIs, and monoclonal antibodies with the aim of ameliorating PDA patients’ quality of life and extend their survival.

## 1. Introduction

Pancreatic Ductal Adenocarcinoma (PDA) is currently the fourth leading cause of cancer-related mortality in the United States and is projected to become the second leading cause of cancer-related deaths by 2030 [1].

Because of the absence of symptoms and the aggressiveness of this malignancy, only 20% of PDA patients are eligible for surgical intervention at the time of diagnosis. The 5-year survival rate for this group of patients in the United States is approximately 13% [2,3].

In order to increase the number of patients eligible for surgery, considerable efforts have been made to identify biomarkers that can facilitate an early diagnosis. Currently, serum carbohydrate antigen (CA) 19-9 and carcinoembryonic antigen (CEA) are employed in the diagnostic assessment of patients with PDA [4]. However, this biomarker is deficient in sensitivity and specificity, which are characteristics associated with a gold-standard marker [4].

The standard-of-care treatment for PDA patients is chemotherapy, which is tailored to the specific stage of the disease and the patient’s overall health status. In fact, the principal regimens are gemcitabine/nab-paclitaxel or FOLFIRINOX (a combination of irinotecan, oxaliplatin, 5-fluorouracil, and leucovorin), which usually confer only a few months of overall survival benefit [5]. Nevertheless, PDA is often resistant to radiotherapy, chemotherapy, and immunotherapy as a consequence of the desmoplastic reaction and the immunosuppressive tumor microenvironment (TME). Histological and molecular studies have revealed the existence of different “subTMEs” that can be linked to a variety of factors, including tumor stage, immune infiltrate, response to treatment, and clinical outcome [6,7]. It is possible to distinguish between two main categories of subTMEs as “reactive” and matrix-rich “deserted”. The former category is characterized by the presence of heterogeneous cancer-associated fibroblasts (CAFs), along with different immune cell subsets, and is associated with a more aggressive basal epithelial phenotype. In contrast, the latter category is characterized by a less prominent presence of activated CAFs and immune cells and is usually associated with resistance to chemotherapy [7]. Notably, in the same tumor, multiple and heterogeneous subTMEs can coexist, which may influence the aggressiveness and responsiveness to therapies [6].

The PDA TME is further characterized by the presence of cytokines, immune cells, hypoxia, a dense fibrotic stroma, and abnormal blood vessels, which contribute to its increased complexity. In particular, interleukins IL-6 and IL-10 are elevated in PDA patients, contributing to in vitro tumor cell invasion by facilitating the proliferation of regulatory T cells (Tregs) and myeloid-derived suppressor cells (MDSCs) [8,9,10,11].

The advent of multiple, complementary approaches such as mass cytometry (CyTOF), single-cell RNA sequencing, and multiplex immunohistochemistry has enabled a more comprehensive characterization of the immune infiltrate. Of note, the PDA milieu is typified by the infiltration of immunosuppressive immune cells, such as M2-polarised macrophages. These have been demonstrated to impede CD8^+^ T cell function and induce Tregs overexpressing programmed death-ligand 1 (PD-L1) on their cell membranes, thereby obstructing the immune response [12].

In contrast to more immunogenic “hot” tumors such as melanoma and renal carcinoma, PDA exhibits low immunogenicity, resulting in a “cold” tumor. This can be ascribed to the very low tumor mutational burden (TMB), with a median of only four mutations identified per megabase and only approximately 1% of the high-TMB PDA [13,14,15]. PDA frequently exhibits activating mutations in the KRAS gene, which drive tumor initiation and growth, and inactivating mutations in tumor suppressor genes such as CDKN2A/p16, TP53, and SMAD4, which contribute to aggressive tumor development [16]. Tumor responsiveness to immunotherapy can be predicted based on the characteristics designated as “hot” or “cold”. The former is associated with a higher level of immune cell infiltration and mutation rate, which determines the expression of new antigens, while the latter is characterized by reduced levels of both factors. Consequently, the expression of tumor-associated antigens (TAAs) and tumor-specific antigens (TSAs) is markedly restricted in PDA [17].

TSAs are exclusively presented on tumor cells, while TAAs are found on both tumor cells and normal cells, but overexpressed in cancer (Peri, Nature Cancer, 2023). Tumor antigens play a critical role in immunotherapy, and understanding their role is essential for developing targeted therapies that improve the efficacy and specificity of treatments. In fact, thanks to the increased availability of sequencing data, current research focuses on neoantigens, which are TSAs derived from genomic alterations and are therefore unique to each patient and not subject to immune tolerance. By focusing on these antigens, immunotherapeutic approaches, such as vaccines, can be more precisely designed, reducing collateral damage to normal cells and increasing the overall efficacy of treatments. 

Enhancing immunotherapy effectiveness in patients with solid tumors requires a dual approach, namely, the modification of the TME and the analysis of immune checkpoint expression on tumor cells, coupled with the identification of biomarkers that predict the response to immune checkpoint inhibitors (ICIs) [18]. Unfortunately, ICI immunotherapy, which targets PD-1/PD-L1 or CTLA-4, has demonstrated potential benefits in only a small subset of PDA patients. Consequently, there have been significant efforts to set clinical trials combining existing immunotherapeutic agents to elicit more effective responses [19].

Vaccines represent a fundamental pillar of immunotherapy because of their capacity to stimulate patient T cells against specific tumor targets, thereby promoting effective antitumor immunity. Vaccine-based clinical trials have demonstrated a different range of antitumor responses in terms of toxicity, survival, and effectiveness across tumor types [20,21]. For example, personalized cancer vaccines have the potential to activate and expand neoepitope-specific T cells, thereby enhancing infiltration [22,23]. The administration of neoantigen vaccines to patients with melanoma was shown to result in the recruitment of a diverse T cell repertoire and a notable increase in CD8^+^ T cell infiltration compared with the levels observed prior to vaccination [24]. It is noteworthy that neoepitope-specific T cells were PD-1, and PD-L1 was upregulated in the TME following vaccination [24,25]. This finding suggests that vaccines provide an appropriate TME for PD-1 inhibitors and can facilitate their induced antitumor immunity.

The use of vaccines in PDA is currently a topic of debate, as the efficacy is limited in time and not all vaccinated patients demonstrate an antitumor response. Consequently, there has been a push to develop more immunogenic target molecules and to enhance the antitumor response by combining vaccination with other therapeutic modalities, such as chemotherapy, radiotherapy, monoclonal antibodies, and ICIs.

The objective of this review is to provide an analysis of recent vaccine-based clinical trials in PDA with the aim of achieving a more robust and long-term immune response by combining different therapeutic strategies.

## 2. Evolution of Vaccine-Based Clinical Trials in Pancreatic Cancer: A Historical Perspective

To analyze the progress and modalities of PDA clinical trials, a dataset registered on clincaltrials.gov until April 2024 was considered (n = 122 NCTids were retrieved). This study employed an automated method for extracting detailed information from the ClinicalTrials.gov database, with a particular emphasis on clinical trials concerning pancreatic cancer vaccines. Data were gathered through a Python script that interfaced with the ClinicalTrials.gov API, using the search term “pancreatic cancer vaccine” to locate relevant studies. This script was developed to identify and classify trials based on various intervention keywords, focusing on all different types of vaccines, along with other related categories like viral vectors, antiangiogenic peptides, and RNA mutations. The filtering process focused on the “ArmsInterventionsModule” section of each trial to pinpoint specific interventions of interest.

**Figure 1:**
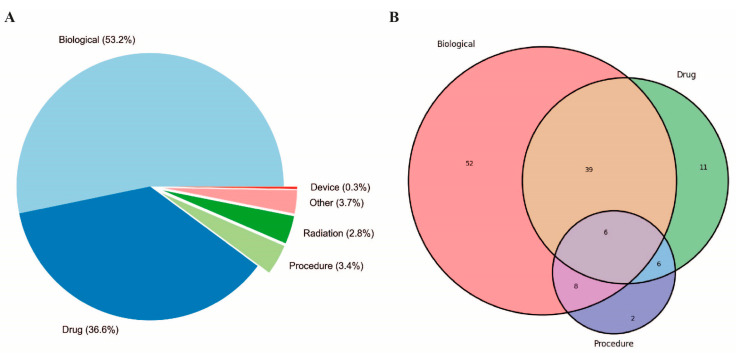
Overview of PDA clinical trials conducted over the past 34 years. (**A**) Distribution of intervention type and (**B**) single and combined approaches for PDA treatment. The percentage (**A**) or the number (**B**) of specific interventions are indicated in the graphs. “Biological” refers to interventions that include clinical trials employing vaccines or antibodies. “Procedures” encompass various medical techniques or surgeries. “Drugs” refers to chemical substances used for treatment, including chemotherapy agents, targeted therapy drugs, and other pharmaceuticals.

**Figure 2:**
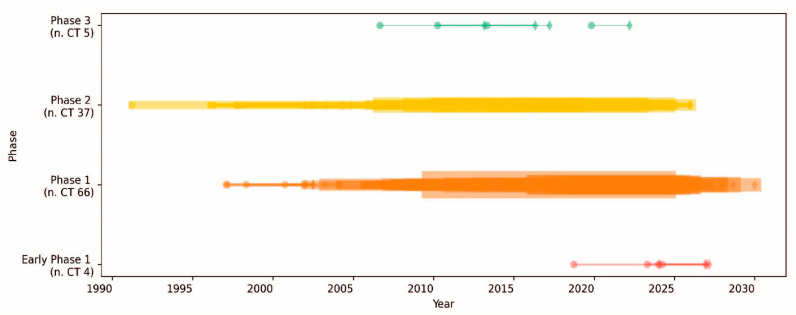
Timeline of clinical trials classified by phase in PDA. The timeline of clinical trials (CTs) over the years is segmented by phase. Each horizontal line represents a clinical trial phase. The start of each phase is marked by a circle, while the end is marked by a diamond. The thickness of the lines is proportional to the number of concurrent CTs conducted. The color coding indicates different phases of clinical trials as follows: early phase 1 (red), phase 1 (orange), phase 2 (yellow), and phase 3 (green). The total number of trials conducted in each phase is shown in brackets.

Moreover, the script, available upon request, generated comprehensive statistics from the extracted data, detailing aspects such as trial phases, study types, availability of results, document classifications, funding sources, and countries where the studies were located. These statistics were derived by systematically examining the “ProtocolSection” of each study and organizing the data into predefined categories. The resultant data provided insights into the clinical trial landscape for pancreatic cancer vaccines, highlighting patterns in trial phases, study methodologies, and geographical distribution. The analysis indicated an increase in the number of approaches and the diversity of therapeutic modalities employed over time, as part of the effort to develop a vaccine-based therapy for PDA (Figure 1). The results of these analyses demonstrated a notable increase in the utilization of combination therapies, which encompass the integration of pharmaceutical agents, biologicals, and surgical procedures (Figure 1 and Figure 2 and Table 1). 

This trend reflects a growing understanding that multi-target approaches may offer more favorable outcomes for treating different cancers, including PDA. Furthermore, more recent personalized approaches, such as the identification of specific TAA and the development of personalized mRNA vaccines (NCT04161755), indicate a shift towards precision medicine tailored to the genetic modification of individual tumors [26]. The development of neoantigen vaccines in combination with ICIs represents a novel therapeutic strategy aimed at enhancing the immune response against cancer cells. This approach has been explored in the context of tumors where the use of ICIs has not resulted in significant improvements in patient survival, such as PDA. Indeed, the most recent trials incorporate advanced technologies, such as next-generation sequencing, to monitor treatment response. For example, in several clinical trials, next-generation sequencing was employed to analyze genetic mutations and track changes in the genetic profile of tumors over time. This integration of cutting-edge technology enabled a more precise and dynamic assessment of patient responses to treatment, thereby demonstrating the advanced capabilities that are being employed in modern clinical research.

**Figure 3:**
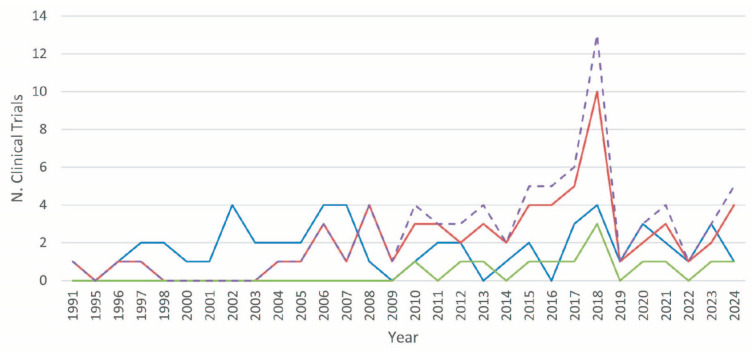
The evolution of PDA clinical trials utilizing vaccines over time. The therapeutic strategies for PDA clinical trials using vaccines are represented as follows: mono-therapy (blue line, includes vaccines only); bi-therapy (red line, combines a vaccine with another strategy, such as drug, device, radiation, or other); tri-therapy (green line, involves a vaccine plus any combination of two different strategies, such as drug, device, radiation, or other); poly-therapy (dashed purple line, total number of trials combining bi- and tri-therapy strategies).

An examination of the annual number of clinical trials conducted with different strategies, including mono- or poly-therapies, from 1991 to 2024, revealed intriguing trends (Figure 3). In the early 1990s, the majority of clinical trials were based on mono-therapies, with occasional bi-therapy trials. The first instances of bi-therapies were observed in 1991 and again in 1996, but they did not become a consistent treatment until the 2000s. During this period, mono-therapies were the preferred option because of their simplicity and established protocols. The early 2000s saw a period of fluctuation in mono-therapies, with peaks in 2002 and 2006, with four trials conducted each year. Bi-therapies started to gain traction around 2004, and there was a significant increase in 2006. In 2010, tri-therapies, a more complex intervention approach, were introduced in clinical trials. This period demonstrated a growing interest and confidence in the utilization of more complex strategies, albeit in limited numbers. From 2011 onwards, bi-therapies experienced a gradual but consistent increase, reaching a peak of 13 trials in 2018. The number of tri-therapies, although less frequent than bi-therapies, demonstrated a consistent presence from 2010 onwards, reflecting a gradual but consistent adoption of this combined strategy. Overall, the data highlight a gradual shift from less complex mono-therapies to more sophisticated bi- and tri-therapies over time.

**Table 1:** Vaccine-based clinical trials for treating pancreatic cancer.

NCT ID	Title	Phase	Treatment	Authors	Sponsor	Publications
NCT02243371	A Randomized Phase 2 Study of the Safety, Efficacy, and Immune Response of GVAX Pancreas Vaccine (With Cyclophosphamide) and CRS-207 With or Without Nivolumab in Patients With Previously Treated Metastatic Pancreatic Adenocarcinoma	2	GVAX vaccine, cyclophosphamide, CRS-207, nivolumab	Dung Le, MD	Sidney Kimmel Comprehensive Cancer Center at Johns Hopkins	Hopkins AC et al. [27].
NCT00836407	A Phase Ib Trial Evaluating the Safety and Feasibility of Ipilimumab (BMS-734016) Alone or in Combination Wit5Allogeneic Pancreatic Tumor Cells Transfected With a GM-CSF Gene for the Treatment of Locally Advanced, Unresectable or Metastatic Pancreatic Adenocarcinoma	1	GVAX vaccine, ipilimumab	Dung Le, MD	Sidney Kimmel Comprehensive Cancer Center at Johns Hopkins	Le DT et al. [28]; Hopkins AC et al. [27].
NCT00084383	A Safety and Efficacy Trial of Lethally Irradiated Allogeneic Pancreatic Tumor Cells Transfected With the GM-CSF Gene in Combination With Adjuvant Chemoradiotherapy for the Treatment of Adenocarcinoma of the Pancreas	2	GVAX vaccine	Daniel A. Laheru, MD	Sidney Kimmel Comprehensive Cancer Center at Johns Hopkins	Lutz E et al. [29].
NCT00389610	A Safety and Efficacy Trial of Vaccine Boosting With Lethally Irradiated Allogeneic Pancreatic Tumor Cells Transfected With the GM-CSF Gene for the Treatment of Pancreatic Adenocarcinoma	2	GVAX vaccine	Daniel A. Laheru, MD	Sidney Kimmel Comprehensive Cancer Center at Johns Hopkins	N/A
NCT02004262	A Phase 2B, Randomized, Controlled, Multicenter, Open-Label Study of the Efficacy and Immune Response of GVAX Pancreas Vaccine (With Cyclophosphamide) and CRS 207 Compared to Chemotherapy or to CRS-207 Alone in Adults With Previously-Treated Metastatic Pancreatic Adenocarcinoma	2B	GVAX vaccine, CRS-207, chemotherapy, cyclophosphamide	N/A	Aduro Biotech, Inc. (San Francisco, CA, USA)	Brockstedt DG et al. [30]; Le DT et al. [31]; Lutz E et al. [29]; Laheru D et al. [32]; Le DT et al. [33]; Le DT et al. [34]
NCT05013216	Mutant KRAS—Targeted Long Peptide Vaccine for Patients at High Risk of Developing Pancreatic Cancer	1	KRAS vaccine, poly-ICLC adjuvant	Nilofer Azad, MD	Sidney Kimmel Comprehensive Cancer Center at Johns Hopkins	N/A
NCT06015724	A Phase 2 Study Evaluating the Efficacy of Anti-CD38 Antibody in Combination With KRAS Vaccine and Anti-PD-1 Antibody in Subjects With Pancreatic Ductal Adenocarcinoma and Refractory Non-Small Cell Lung Cancer	2	KRAS vaccine, daratumumab, nivolumab	Samir Khleif, MD	Georgetown University	N/A
NCT04117087	Pooled Mutant KRAS-Targeted Long Peptide Vaccine Combined With Nivolumab and Ipilimumab for Patients With Resected Mismatch Repair Protein (MMR-p) Colorectal and Pancreatic Cancer	1	KRAS vaccine, nivolumab, ipilimumab	Neeha Zaidi, MD	Sidney Kimmel Comprehensive Cancer Center at Johns Hopkins	N/A
NCT02261714	A Phase I/II Trial of TG01 and Gemcitabine as Adjuvant Therapy for Treating Patients With Resected Adenocarcinoma of the Pancreas	1/2	KRAS vaccine	Daniel PALMER, Juan VALLE, Svein DUELAND, Yuk Ting MA, Emiliano Calvo	Targovax ASA (Norway)	Palmer DH et al. [35]
NCT00358566	“Primovax”—A Phase III Trial Comparing GV1001 and Gemcitabine in Sequential Combination to Gemcitabine Monotherapy in Advanced Un-Resectable Pancreatic Cancer.	3	telomerase vaccine, gemcitabine	Ask Aabenhus, MSc.	Pharmexa A/S (Hørsholm, Denmark)	N/A
NCT00425360	A Prospective, Phase III, Controlled, Multicentre, Randomised Clinical Trial Comparing Combination Gemcitabine and Capecitabine Therapy With Concurrent and Sequential Chemoimmunotherapy Using a Telomerase Vaccine in Locally Advanced and Metastatic Pancreatic Cancer [TELOVAC]	3	telomerase vaccine, sargramostim, capecitabine, gemcitabine hydrochloride	Gary W. Middleton	Royal Liverpool University Hospital	N/A
NCT00622622	Phase I Study of Gemcitabine With Antiangiogenic Vaccine Therapy Using Epitope Peptide Restricted to HLA-A*2402 Derived From VEGFR2 in Patients With Unresectable, Locally Advanced, Recurrent or Metastatic Pancreatic Cancer	1	VEGFR-2 vaccine, gemcitabine	Hiroki Yamaue, MD	Wakayama Medical University	Wada S et al. [36]; Li Y et al. [37]; Niethammer AG et al. [38]; Date Y et al. [39]; Correale P et al. [40]; Miyazawa M et al. [41]
NCT01486329	VXM01 Phase I Dose Escalation Study in Patients With Locally Advanced, Inoperable and Stage IV Pancreatic Cancer to Examine Safety, Tolerability, and Immune Response to the Investigational VEGFR-2 DNA Vaccine VXM01	1	VEGFR-2 vaccine	Thomas Schmidt, MD	Vaximm GmbH (Mannheim, Germany)	Niethammer AG et al. [42]
NCT03645148	Safety, Tolerability and Partial Efficacy Study of a Personalized Neoantigen Cancer Vaccine in Treating Patients With Advanced Pancreatic Cancer	1	neoantigen vaccine, GM-CSF	N/A	Zhejiang Provincial People’s Hospital	Weden S et al. [43]; Chen Z et al. [44]
NCT04161755	Phase 1 Clinical Trial of Personalized Neoantigen Tumor Vaccines and Programmed Death-Ligand 1 (PD-L1) Blockade in Patients With Surgically Resected Pancreatic Cancer	1	neoantigen vaccine, atezolizumab, mFOLFIRINOX	Vinod Balachandran, MD	Genentech, Inc.(San Francisco, CA, USA)	N/A
NCT00203892	A Randomized Pilot Phase II Study of Immunization With Modified CEA (CAP1-6D) Peptide In Patients With Locally Advanced Or Surgically Resected Adenocarcinoma of the Pancreas	1/2	neoantigen vaccine	Hedy Kindler, MD	University of Chicago	Geynisman DM et al. [45]
NCT03662815	Safety, Tolerability and Partial Efficacy Study of a Personalized Neoantigen Cancer Vaccine in Treating Patients With Advanced Malignant Tumor	1	neoantigen vaccine, GM-CSF	N/A	Sir Run Run Shaw Hospital	Shou J et al. [46]; Fang Y et al. [47]

## 3. Synergistic Strategies: Advancing Pancreatic Cancer Treatment through Combined Therapies

Surgery, radiotherapy, and chemotherapy are the standard-of-care treatment options for PDA patients [48]. However, tumors are typically beyond the scope of surgical resection in advanced stages, and the benefits of radiotherapy and chemotherapy are limited [49,50,51,52,53]. In the resected setting, the standard adjuvant treatment is usually the modified FOLFIRINOX (mFOLFIRINOX: fluorouracil, irinotecan, oxaliplatin) chemotherapy regimen, which has been associated with an overall survival of 54 months [54] and survival in the locally advanced setting, even with chemotherapy (FOLFIRINOX or gemcitabine plus nab-paclitaxel) and radiation therapy, which has been observed to be approximately 2 years [55,56]. In the metastatic setting, the same regimens of FOLFIRINOX, gemcitabine plus nab-paclitaxel, or with the recently introduced NALIRIFOX (fluorouracil, liposomal irinotecan, and oxaliplatin), have a median overall survival of less than 12 months [54,57,58]. With regard to subsequent lines of therapy, there is currently no established standard of care. Consequently, both chemotherapy and enrolment in clinical trials may be considered potential avenues for treatment if the patient’s performance status is sufficient [53]. Chemotherapy is able to alter the composition of the tumor immune microenvironment [59]; it inhibits the generation of immunosuppressive immune cells, such as Tregs, MDSCs, and tumor-associated macrophages, and promotes a more inflammatory immune infiltrate [60,61]. Additionally, chemotherapy induces tumor cell death, leading to increased presentation of neoantigens [62]. In addition, an increase in CD4 and CD8 T cells and a decrease in FoXP3 Treg cells was observed in the TME of PDA patients after chemotherapy [59]. The alteration of TME after conventional chemotherapy creates opportunities for concurrent or sequential immunotherapy [59].

Radiotherapy represents a crucial therapeutic modality for PDA, as it induces an immune response that enhances the immunogenicity of the tumor [63,64]. The objective of conventional radiotherapy is to achieve the highest possible radiation dose to the tumor site while simultaneously minimizing damage to surrounding normal tissue [65]. Clinical studies have demonstrated that radiotherapy is an efficacious cancer treatment, as it renders vulnerable cancer cells susceptible to immune system attack, thereby inducing tumor regression in non-irradiated areas (abscopal effect) [66,67,68,69,70]. Radiotherapy exerts dose- and lineage-dependent effects on immune cell survival, migration, activation, and proliferation within the TME. Furthermore, radiotherapy induces phenotypic alterations that modulate the immune susceptibility of tumor cells. This has created interest in the potential of radiotherapy for enhancing the efficacy of immunotherapies. However, in order to realize the potential of such combinations, it is important to understand how best to deliver radiotherapy in order to achieve the desired activation of immunological mechanisms [71]. Radiotherapy induces the following effects: (i) activating a type 1 interferon (IFN) antitumor response, (ii) favoring the expression of MHCI on tumor cells that enables tumor antigen presentation and tumor cell recognition by effector T cells, (iii) promoting greater tumor cell plasma membrane expression of death receptors (such as Fas and Dr5) and inflammatory protein markers of immunogenic cell death (such as calreticulin) [72,73,74], and (iv) favoring tumor cell surface PD-L1 expression, leading to changes in IFNβ and IFNγ production in the TME [75,76,77]. Moreover, the TME composition and the activation of the immune cell infiltrate observed in a tumor after radiation are affected by the composition of the TME prior to radiation, the radiation dose, the diverse radiosensitivity of immune lineages present at the time of radiotherapy, and the distinct response of circulating and resident immune lineages to the inflammatory effects induced by radiotherapy [71]. Radiotherapy has been demonstrated to induce the release of cytokines that facilitate the polarization or recruitment of immunosuppressive cells, thereby reinforcing the immunosuppressive characteristics of the TME [63]. The combination of radiotherapy and immunotherapy has been shown to have a synergistic antitumor effect, resulting in improved outcomes for patients with non-small-cell lung cancer [78]. In contrast, comparable outcomes have not been achieved in other solid tumors, such as PDA, where the immunosuppressive TME represents the most significant barrier to effective immuno- and radio combination therapy [63]. 

## 4. Immune Checkpoints Inhibitors

Immune checkpoints are signaling molecules that regulate the response of T cells on antigen presentation. They can either stimulate or inhibit this response [18,79,80,81,82]. The ligand is often found on the antigen-presenting cell, whereas its corresponding receptor is typically localized on the membrane of T cells [81]. Inhibitory molecules include programmed cell death protein 1 (PD-1), programmed cell death-ligand 1 (PD-L1), programmed cell death-ligand 2 (PD-L2), cytotoxic T-lymphocyte-associated antigen 4 (CTLA-4), CD86, and CD80 [81,83]. CTLA-4 is an immune checkpoint receptor expressed on Treg cells and activated conventional T cells. It functions as a negative regulator of T cell activation. The ligands of CTLA-4 are CD80 and CD86, which are expressed on antigen-presenting cells and provide costimulatory signals to T cells [84,85]. Since CTLA-4 has a higher affinity for CD80 ligands than CD28, CTLA-4 ligation delivers an inhibitory signal to T cells, whereas CD28 ligation delivers a stimulatory signal [86,87]. The therapeutic success of targeting CTLA-4 with a human anti-CTLA-4 antibody for treating melanoma has been demonstrated [88,89], suggesting that blocking CTLA-4 may represent a promising new approach for cancer therapy. This strategy represents a novel approach to inducing host responses against tumors, as evidenced by the survival rate increase observed in melanoma patients [90,91] and lung cancer patients [92,93] treated with a combination of PD1 and CTLA-4 inhibition. PD-1 protein, known as another immune checkpoint, is expressed on the surface of activated T cells and is associated with programmed cell death [94]. PD-1, which is overexpressed in a multitude of tumors [85], together with one of its ligands, PD-L1 can suppress the overstimulation of immune responses and instead promote the maintenance of immune tolerance to self-antigens [95]. Similarly, PD-L1 is expressed by immune cells (including T cells, B cells, macrophages, dendritic cells (DCs), and mast cells) as well as various cancer cells, including those of the breast, cervix, colorectum, stomach, glioblastoma, melanoma, non-small-cell lung, ovary, PDA, and urinary bladder [96,97]. The binding of PD-1 to its ligands has been demonstrated to inhibit T cell activity, thereby restricting tumor cell killing [98,99] and preventing autoimmunity [100]. In preclinical models, blocking the ligation between PD-1 and PD-L1 using highly specific antibodies has been demonstrated to augment the antitumor immune response in vitro and to destroy cancer cells, thereby restoring T cell function [97,101,102]. The other ligand of PD1, PDL2, is primarily expressed in tumor cells, macrophages, DCs, and mast cells [103]. The expression of PD-L1 is more widespread among tumor cells than that of PD-L2, and antibodies that target PD-L1 display superior clinical efficacy compared with those that target PD-L2. Consequently, the majority of cancer immunotherapy studies have focused on the inhibition of the interaction between PD-1 and PD-L1 [104]. 

Ipilimumab, a CTLA-4 antibody, was the first immune checkpoint inhibitor to be approved by the U.S. Food and Drug Administration (FDA) for treating patients with metastatic melanoma in 2011 [105]. Nevertheless, PD-1/PD-L1 antibodies (nivolumab, pembrolizumab, atezolizumab, durvalumab, and avelumab) rapidly surpassed this treatment option because of their favorable response rates and relatively low toxicity [106]. Antibodies targeting the PD-1/PD-L1 axis have been approved for treating numerous cancers, including non-small-cell lung cancer, small-cell lung cancer, renal cell carcinoma, head and neck squamous cell carcinoma, urothelial carcinoma, breast cancer, and Hodgkin’s lymphoma [107]. However, PDA stands out as a cancer that does not respond to ICI-based immunotherapy [108,109,110]. To improve the negative results obtained in clinical trials with ICIs in PDA patients, and given the growing evidence that a single therapeutic approach is insufficient to halt neoplastic progression, a decision was made to combine ICI therapy or chemo-radiotherapy treatments with alternative strategies, with a particular emphasis on vaccination.

Significant advancements have been made over the past decade in the utilization of cancer vaccines for solid tumors [111], representing an area of extensive investigation within tumor immunotherapy research, particularly for PDA [112,113]. In this context, tumor progression results in a gradual weakening of the monitoring and recognition ability of the immune system, which allows tumor cells to evade immune surveillance [49]. It has been demonstrated that tumor vaccines can facilitate the expansion, amplification, and activation of tumor-specific T/B cells via active immunization [114], thereby enhancing the immune response against tumors and enabling the specific eradication of tumor cells. 

Given these meager survival data, numerous trials have been conducted with novel targets in an effort to enhance overall survival and response rates in patients.

## 5. GVAX-Based Vaccines

Granulocyte–Macrophage Colony-Stimulating Factor (GM-CSF)-secreting allogeneic pancreatic tumor cell (GVAX) immunotherapy consists of two irradiated human allogeneic pancreatic tumor cell lines (CG8020/CG2505) that underwent modification to enable the secretion of GM-CSF (NCT00389610). This is a cytokine that induces the maturation of DCs. GVAX is a versatile source of tumor antigens, stimulating a diverse range of T cell responses that diversify to multiple tumor antigens shared between the vaccine and the patient’s tumors [115]. The vaccine was developed based on the findings of preclinical studies that demonstrated the necessity of high levels of GM-CSF secretion at the site of vaccination for several days to elicit effective antitumor immune responses by anticancer vaccines [116]. The expression of GM-CSF by cancer cells has been demonstrated to prime the immune system with great efficiency, as it enhances the capacity of DCs to present tumor antigens [117].

In 2001, Jafee and colleagues conducted the first clinical trial utilizing GVAX in 14 PDA patients with stage 1, 2, or 3 tumors [118]. The GVAX vaccine was found to be safe in patients and to induce dose-dependent systemic antitumor immunity, as evidenced by increased post-vaccination delayed-type hypersensitivity responses against autologous tumors [118]. In order to enhance the efficacy of this therapeutic approach, clinical trials were conducted, in which the GVAX vaccine was administered in combination with chemo- and/or radiotherapy regimens and ICIs.

In the Phase II clinical trial NCT00084383, patients with resected stage I or II adenocarcinoma were treated with the GVAX vaccine administered 6–8 weeks after surgery. At 4–8 weeks following the completion of the final cycle of 5-FU-based chemoradiation, eligible patients received three additional vaccinations at 1-month intervals. Patients who continued to remain disease-free received a fifth “booster” vaccination 6 months following the fourth vaccination [29]. The study demonstrated that (i) the administration of up to five repetitive treatments with the GVAX vaccine was well tolerated when sequenced with adjuvant chemoradiation; (ii) the treated patients reported overall survivals ranging from 15 to 20 months [119,120,121,122,123,124,125,126,127]; and (iii) the induction and maintenance of enhanced post-immunotherapy mesothelin-specific T cell responses were associated with prolonged disease-free survival in the HLA-A*01 and HLA-A*02 patients. The results of the study confirmed those of the phase I study, indicating that the post-immunotherapy induction of mesothelin-specific CD8+ T cells correlated with improved disease-free survival [118,128]. In addition, the analyses were extended to encompass multiple HLA-A*01:01 and HLA-A*02:01 epitopes derived from mesothelin [29]. A preliminary investigation was conducted at the Sidney Kimmel Comprehensive Cancer Center at Johns Hopkins and the Mary Crowley Medical Research Center, in which GVAX was utilized as an immunotherapy agent, either as a standalone treatment or in combination with cyclophosphamide (Cy), in patients diagnosed with advanced PDA [32]. The administration of GVAX alone or in combination with Cy elicited minimal treatment-related toxicity in patients with advanced PDA. It is noteworthy that the average survival rate for patients with PDA treated with GVAX alone was 2.3 months, while the average survival rate for patients treated with GVAX + Cy was 4.3 months [32]. In an effort to improve this therapeutic approach, a phase IIb study (NCT02004262, ECLIPSE Study) was conducted, combining the GVAX vaccine with Cy and CRS-207 (live, attenuated Listeria monocytogenes expressing mesothelin) [30,31,33,34]. Patients with previously treated metastatic PDA were randomly assigned to receive Cy/GVAX+ CRS-207, CRS-207, or a physician’s choice of single-agent chemotherapy. Cy was administered via intravenous infusion on the first day of weeks 1 and 4. The GVAX vaccine was administered by intradermal injection on the second day of weeks 1 and 4. CRS-207 was administered via intravenous infusion on the first day of weeks 7, 10, 13, and 16. The study did not achieve its primary efficacy endpoint, showing no statistically significant difference between the Cy/GVAX + CRS-207 and single-agent chemotherapy groups. Moreover, the most commonly reported adverse events across all treatment groups were chills, pyrexia, fatigue, and nausea. It is noteworthy that no deaths were attributed to the treatment [34]. 

A triple therapy was also proposed. The GVAX vaccine was administered in locally advanced, unresectable, or metastatic PDA patients in combination with ipilimumab (targeting CTLA-4 (NCT00836407) or nivolumab (targeting PD-1) as well as CRS-207 (NCT02243371). In the clinical trial NCT00836407, ipilimumab was administered intravenously on weeks 1, 4, 7, and 10. The GVAX vaccine was administered intradermally on weeks 1, 4, 7 and 10. Both therapies were repeated at 12-week intervals. Two patients treated with ipilimumab exhibited evidence of stable disease, yet none demonstrated a biochemical response, as indicated by CA19-9 [28]. In contrast, three patients treated with the GVAX vaccine exhibited prolonged disease stabilization, while seven patients demonstrated a decline in CA19-9 [28]. In two of these patients, disease stabilization was observed subsequent to an initial period of progression. Median overall survival and 1-year overall survival were both superior in patients treated with the GVAX vaccine [28]. In addition, the studies demonstrated that (i) the majority of patients who underwent these treatments exhibited a net diversification of their peripheral TCR repertoires; (ii) patients receiving ipilimumab experienced more pronounced alterations in TCR repertoires, particularly when it was administered in combination with GVAX; and (iii) both low baseline clonality and a high number of expanded clones following treatment were associated with significantly longer survival in patients who received ipilimumab. However, this was not observed in those who received nivolumab [27]. These therapies have been observed to exert quantifiable effects on the peripheral TCR repertoire, which align with their proposed mechanisms of action. This indicates the possibility that TCR repertoire profiling may serve as a biomarker of clinical response in patients with PDA who are undergoing immunotherapy. Interestingly, this analysis demonstrated that substantial alterations in the T cell repertoire can be discerned in PDA tumors, which are typically regarded as immunologically suppressed, and employed to anticipate clinical outcomes [27,129]. 

In the clinical trial NCT02243371, patients with metastatic PDA who previously underwent treatment were divided into two groups. One group received a Cy/GVAX vaccine/CRS-207/nivolumab combination (arm 1), while the other received a Cy/GVAX vaccine/CRS-207 mono-therapy (arm 2). Patients enrolled in arm 1 received CRS-207 intravenously on day 2 of cycles 3–6, nivolumab intravenously on day 1 of cycles 1–6, the GVAX vaccine in six intradermal injections on day 2 of cycles 1 and 2, and Cy administered intravenously on day 1 of cycles 1 and 2. Patients in arm 2 received CRS-207 intravenously on day 1 of cycles, the GVAX vaccine administered in six intradermal injections on day 2 of cycles 1 and 2, and Cy administered intravenously on day 1 of cycles 1 and 2. The median overall survival time was 5.88 months in arm 1 and 6.11 months in arm 2, while the median progression-free survival was 2.23 months (arm 1) and 2.17 months (arm 2).

The combination of several drugs with the GVAX vaccine represents a promising strategy for developing novel immunostimulatory strategies in patients with PDA.

## 6. RAS- and KRAS-Targeting Vaccines

Developing vaccines targeting RAS and KRAS mutations represents a significant advance in the field of personalized cancer treatments, with the aim of improving the survival rates of PDA patients by harnessing the immune system to specifically recognize and target tumor cells that harbor these mutations. The RAS family of genes includes the following three members: HRAS, NRAS, and KRAS. RAS genes encode proteins involved in cell signaling pathways that regulate cell growth and division [130]. Among these, KRAS mutations are particularly prevalent in PDA, occurring in over 90–92% of cases [130]. When mutated, KRAS becomes oncogenic, leading to uncontrolled cell division and contributing to PDA aggressiveness [131]. Vaccines designed to target these mutations are developed by first identifying the specific mutations present in the patient’s tumor through genetic sequencing. Subsequently, synthetic peptides or proteins are engineered to mimic the mutated segments of the KRAS protein. The peptides are employed as antigens to elicit a specific immune response against cancer cells that exhibit the aforementioned mutations.

Different types of vaccines have been developed, such as peptide-based vaccines constituted by short peptides corresponding to the mutated regions of KRAS and DNA/RNA vaccines that deliver genetic material to trigger an immune response. Another approach involves DC-based vaccines, in which DCs are loaded with KRAS/RAS peptides to prime the immune system against cancer cells [132], leading to the activation of cytotoxic T cells and memory cells and thereby providing long-term surveillance against tumor recurrence [133].

In April 2022, a phase 1 clinical trial (NCT05013216) based on a mutant KRAS-targeted long peptide vaccine combined with the adjuvant poly-ICLC was initiated at the Sidney Kimmel Comprehensive Cancer Center at Johns Hopkins for patients at high risk of developing PDA and pancreatic cystic neoplasm. Patients at high risk of developing pancreatic cancer were administered a KRAS peptide vaccine with a poly-ICLC adjuvant on weeks 1, 3, and 5 of the initial treatment phases. Boost vaccinations were administered on week 13. All participants returned to the study facility approximately 28 days after their final vaccination for an end-of-treatment and safety evaluation. Patients with evidence of a pancreatic cystic neoplasm received a KRAS peptide vaccine with a poly-ICLC adjuvant as two prime vaccinations, administered on weeks 1 and 2. The decision to proceed with surgical intervention was at the discretion of the treating hepatobiliary surgeon. Patients were required to return to the study site after approximately week 4 for a safety evaluation prior to undergoing surgery. At the conclusion of the treatment period, which occurred on study week 8, patients underwent an end-of-treatment visit. The trial was designed to assess the safety of the vaccine and to determine whether it could expand the number of IFN-γ-producing CD8+ and CD4+ T cells specific to mutant KRAS in patients at high risk of developing PDA [134]. The clinical trial is ongoing and will be completed in 2026.

A total of 23 PDA patients who received the vaccine following surgical resection were monitored for over a decade in order to gain insight into their long-term immunological T cell reactivity and survival. It was observed that 85% of patients who received the vaccine responded immunologically to the administered therapy. The median survival period for these responders was 28 months, compared with 27.5 months for non-responder patients. Strikingly, the 10-year survival rate was 20% compared with zero in a cohort of non-vaccinated patients. Of note, three patients mounted a memory response up to 9 years after vaccination, emphasizing the potential of mutant KRAS vaccines [43]. 

The pooled mutant KRAS-targeted long peptide vaccine in combination with nivolumab (anti-PD-1) and ipilimumab is a recent phase I study that is still recruiting (NCT04117087). The study will enroll patients who underwent resection of a PDA following neoadjuvant and/or adjuvant chemotherapy and/or radiation therapy. Additionally, patients with metastatic colorectal cancer who underwent treatment with two or more lines of chemotherapy will be included. The KRAS peptide vaccine will be administered on days 1, 8, and 15 of cycle 1 and on day 1 of cycle 2. Subsequent boost vaccinations will be administered on 28-day intervals. Nivolumab will be administered via intravenous infusion over a period of 30 min on the first day of each 21-day cycle. The booster phase will be administered every 28 days, commencing with cycle 5. Ipilimumab will be administered as a 30 min intravenous infusion on day 1 of cycles 1 and 3 of the study. The trial, which is being conducted by the Sidney Kimmel Comprehensive Cancer Center at Johns Hopkins, aims to demonstrate that the combination therapy elicits robust T cell responses against mutant KRAS epitopes, which may ultimately lead to enhanced disease-free and progression-free survival [135,136]. 

In order to enhance the efficacy of the KRAS vaccine and anti-PD-1 treatment, an anti-CD38 antibody (daratumumab) is being administered with the objective of facilitating the killing of cancer cells by the immune system [137]. The objective of this ongoing phase 2 study (NCT06015724), conducted by Georgetown University, is to evaluate the safety and efficacy of this approach with the aim of activating both major histocompatibility complex (MHC) class II-restricted CD4 helper T cells and MHC class I-restricted CD8 cytotoxic T cells [43,138,139]. This study will be conducted in patients with advanced non-small cell lung cancer or PDA patients who failed one prior treatment. A further study demonstrated the safety and efficacy of Targovax TG01 (a KRAS-targeted vaccine)/GM-CSF in combination with gemcitabine in a phase 1/2 trial (NCT02261714) in resected stage I or II PDA patients [35]. TG01 and GM-CSF were administered on days 1, 8, 15, 22, and 36. Gemcitabine was administered at least 3 weeks after TG01/GM-CSF and was administered on days 1, 8, and 15 of a 4-week cycle for up to six cycles in total. Following the conclusion of chemotherapy, the administration of GM-CSF and TG01 injections resumed at 4-week intervals, extending from the end of the chemotherapy period until week 52, and then at 12-week intervals from week 52 until week 104. TG01 and GM-CSF were administered intradermally, while gemcitabine was administered intravenously over a period of 30 min. In the event that patients were unable to start TG01 quickly after surgery, the vaccination started at the same time as the chemotherapy as long as they started within 12 weeks of surgery. Gemcitabine started at the same time as TG01/GM-CSF and was given on days 1, 8, and 15 of a 4-week cycle for up to six cycles in total. It is noteworthy that following the treatment of 19 patients, a modified TG01/GM-CSF dosing regimen was introduced. The objective was to ascertain whether the absence of vaccinations during chemotherapy treatment could elicit comparable immune responses while enhancing the safety profile, particularly with regard to the allergic reactions observed in the previous cohort [35]. In light of these findings, a further 13 patients received a modified vaccination schedule with a reduced antigen burden, with no serious adverse events related to TG01 [35]. A positive immune response was observed in 95% of patients in the main cohort and 92% in the modified cohort. The median overall survival for the main cohort was 33.1 months, while the median disease-free survival was 13.9 months. For the modified cohort, the median overall survival was 34.3 months, with a median disease-free survival of 19.5 months [35]. In addition, a total of 18/19 (95%) and 12/13 (92%) patients had a positive immune response in the main and modified cohorts, respectively [35]. In light of the findings of the study, the use of combination chemotherapy (gemcitabine + nab-Paclitaxel or modified FOLFIRINOX) in the adjuvant setting, in combination with a safe, well-tolerated, non-cytotoxic agent such as TG01, demonstrated encouraging safety and immunological and enhanced survival rates [35].

## 7. Neoantigen-Based Vaccines

In cancer, the genome is characterized by a high degree of instability, with tumor cells exhibiting a proclivity for frequent mutations and considerable heterogeneity. The expression of non-synonymous mutations resulted in the generation of neoantigens, which are non-autologous proteins with individual specificity [140]. In contrast to TAAs, which are also expressed by normal cells, neoantigens are tumor-specific and distinguished by a stronger immunogenicity, a greater affinity for MHC, and notably are not subjected to central immune tolerance. Tumor neoantigens are capable of being recognized by CD8 and CD4 T cells within the tumor environment, thereby eliciting an antitumor immune response in vivo [141,142,143]. The concept of employing neoantigens as vaccines to stimulate the patient’s immune system actively against the tumor has gained recognition. 

PDA is not characterized by a high TMB; therefore, identifying mutations and predicting neoantigens represent crucial steps in the formulation of an effective vaccine. It is estimated that only 10% of non-synonymous mutations in tumor cells are able to generate mutant peptides with high MHC affinity [144]. Furthermore, only 1% of peptides with high MHC affinity are capable of being recognized by T cells [145]. It is evident that despite the occurrence of over 30,000 mutations in the PDA genome and the prediction of hundreds of epitopes able to trigger an immune response, the number of neoantigens that have been proven to elicit an effective antitumor response is very limited. This suggests that both the quantity and the quality of neoantigens play a crucial role in the development of an effective immunotherapeutic strategy [146,147]. 

Despite the promising results observed thus far, several challenges impede the widespread application of neoantigen-based vaccines in PDA. These include the immunosuppressive TME and high genetic heterogeneity, which limit the identification of universally applicable neoantigens [148]. Indeed, apart from driver genes, the majority of mutations are shared among patients in less than 20% of cases [149], and the probability of different individuals developing the same neoantigens is extremely low [150]. Furthermore, the necessity for biopsy in order to sequence the genome of cancer cells represents a significant challenge. It is important to note that the sequenced piece may differ from other parts of the tumor, which could result in the omission of crucial mutations. To address the challenge of acquiring tumor genomes, researchers are exploring the potential of circulating free DNA (cfDNA) or circulating tumor cells (CTCs) as alternative sources [151,152,153,154]. 

Several strategies are currently being investigated to increase the therapeutic potential of neoantigen-based vaccines, including the combination with ICIs to overcome immune suppression and improve the accuracy of neoantigen prediction with advanced computational methods. Lastly, novel delivery systems, including nanoparticle-based and viral-vector-based platforms, are being investigated to enhance the immunogenicity of neoantigen vaccines.

An early-phase clinical trial (NCT03645148) was conducted to assess the safety, tolerability, and immunogenicity of a personalized neoantigen peptide vaccine in seven Chinese patients with advanced PDA. Each patient’s vaccine (four peptides given on days 1, 4, 8, 15, 22, 78, and 162 for a total of seven doses) was customized based on the specific neoantigens identified from their tumor mutations. In patients who received the personalized neoantigen peptide vaccine, no severe adverse effects were observed. The mean overall survival associated with vaccine treatment and progression-free survival was reported to be 24.1, 8.3, and 3.1 months, respectively [44]. A higher presence of effector memory CD4 and CD8 T cells was observed in the peripheral blood. In addition, the vaccine was able to expand antigen-specific TCR clones, indicating its potential involvement in the activation of a specific subset of T cells able to kill cancer cells [44]. 

A phase 1/2 clinical trial (NCT03662815) was designed to evaluate the safety, tolerability, and preliminary efficacy of a personalized neoantigen vaccine, which was previously employed in NCT03645148 in combination with GM-CSF in patients with metastatic PDA. The peptide vaccine was administered subcutaneously on days 1, 4, 8, 15, 22, 78, and 162 for a total of seven doses and every 2 to 3 months afterward until disease progression was found, while GM-CSF was administered 30 min before peptide vaccine. In addition, ten patients received radiofrequency ablation treatment within 6 months before vaccination (Cohort 1), and the remaining 18 patients did not (Cohort 2). The median progression-free survival and median overall survival were observed to be longer in patients in Cohort 1 than in patients in Cohort 2 (4.42 and 20.18 months vs. 2.82 and 10.94 months). The ex vivo IFN-γ ELISpot assay demonstrated that patients in Cohort 1 exhibited more robust neoantigen-specific immune responses at baseline and post-vaccination [46,47].

A phase 2 clinical trial (NCT00203892) aimed to validate the therapeutic potential of a modified CEA peptide in combination with GM-CSF in PDA patients who underwent chemo- and radiation therapy, HLA-A2+ and CEA-expressing. In fact, CEA is expressed in over 90% of PDA patients, but it is poorly immunogenic because of immune tolerance; thus, the idea was to set a vaccination strategy based on the identification of CEA-related neoantigens. The vaccine containing the modified CEA peptide, Montanide ISA-51, and GM-CSF was administered on day 1 of each 14-day cycle until progressive disease or dose-limiting toxicity for a maximum of 24 cycles. No instances of toxicity were documented, and a T cell dose-dependent response was evidenced [45]. A T cell response was observed in 100% of patients who received the maximum dose of the vaccine. A total of 37% of patients who received treatment exhibited stable disease, while the median overall survival for the entire cohort of 19 patients was 334 days, with a median progression-free survival of 56 days. Overall, 37% of patients were alive at a minimum of 32 months from the initiation of the trial. Of the 17 patients with locally advanced or metastatic disease, five (29%) remained alive [45].

A recent phase 1 clinical trial (NCT04161755) [26] evaluated the therapeutic potential of the combination of a personalized neoantigen-based vaccine with a PD-L1 blocker (atezolizumab) and mFOLFIRINOX-based chemotherapy in surgically resected PDA patients, aiming to enhance the ability of the immune system to counteract the progression of PDA. The vaccine targets up to 20 neoantigens identified from the genetic profile of each patient’s tumor and was delivered as RNA molecules. Atezolizumab was administered 6 weeks post-tumor resection, the vaccine was delivered at 9 weeks post-tumor resection, and mFOLFIRINOX was administered 21 weeks post-tumor resection. The study found that the vaccine successfully stimulated T cells to recognize cancer cells, leading to an expansion of neoantigen-specific T cell clones. The combined therapy was generally well-tolerated, with manageable side effects, indicating that this strategy is feasible and promising for surgically resected PDA patients.

## 8. Telomerase and Anti-Angiogenic Vaccines

Telomerase is expressed in 85–90% of PDA cases and may represent a potential target for active cancer immunotherapy [155]. 

GV1001, a peptide derived from the active site of the human telomerase reverse transcriptase, is a widely expressed TAA and a potentially applicable target for anticancer immunotherapeutic strategies [156]. GV1001 regulates the vascular endothelial growth factor (VEGF)-A-stimulated signaling network, including the FAK, Src, MEK, ERK, and AKT pathways; conversely, suppression of this regulation induces the expression of VEGF receptor 2 (VEGFR-2) and Matrix Metalloproteinase (MMP)-2 [157]. The results demonstrate that GV1001 has the capacity to suppress the proliferation and invasion of non-small cell lung cancer cells and the release of VEGF from these cells. This suggests that GV1001 may play a regulatory role in tumor-derived angiogenesis and the growth and proliferation of cancer cells [157]. 

The median overall survival times were 7.89, 6.94, and 8.36 months for arms 1, 2, and 3, respectively. A total of 48 patients with non-resectable PDA received the GV1001 vaccine in combination with GM-CSF. The vaccine was administered by intradermal injection in accordance with the prescribed schedule, comprising three injections in week 1 and one weekly injection in weeks 2, 3, 4, 6, and 10. Three distinct doses of vaccine were administered in low, intermediate, and high doses. From 5 to 15 min before each vaccine injection, 3GM-CSF was injected intradermally at the vaccination site. Monthly booster vaccinations were offered for up to 1 year. The follow-up treatment was given to patients in good general health [158]. Immune responses were measured as delayed-type hypersensitivity skin reactions and in vitro T cell proliferation. GV1001 was well tolerated. Immune responses were observed in 24 of 38 evaluable patients, with the highest rate (75%) in the mid-dose group. Median survival was 8.6 months in the mid-dose group and significantly longer in the low-dose and high-dose groups. The 1-year survival rate for evaluable patients in the mid-dose arm was 25% [158]. The results show that GV1001 is immunogenic and safe to use. The survival data suggest that induction of an immune response correlates with prolonged survival and that the vaccine may offer a new treatment option for PDA patients, encouraging further clinical trials [158]. This study established the rationale for evaluating the enhanced efficacy of GV1001 in combination with GM-CSF and gemcitabine in non-resectable PDA patients with a life expectancy of at least 3 months [155]. The 16-amino acid hTERT peptide (EARPALLTSRLRFIPK) of GV1001 was administered in 0.10 mL saline (groups A/B) and in 0.20 mL saline (group C). Patients in group A received GV1001 intradermally on days 1, 3, and 5 in the first week, followed by a once-weekly schedule in weeks 2, 3, 4, and 6. At each vaccination, patients also received GM-CSF intradermally 15 min prior to GV1001 at the vaccination site. Group B received the same vaccination schedule as group A, except that GM-CSF was administered intradermally for 5 consecutive days in the first week (days 1–5) and GM-CSF for 4 consecutive days in weeks 2, 3, 4, and 6 starting on the day of peptide vaccination. Gemcitabine was given intravenously once a week for 7 consecutive weeks in both groups. If gemcitabine and vaccine were given on the same day, the vaccine was given first. In groups A/B, after the initial 7 weeks, gemcitabine was continued until disease progression at the clinician’s discretion. In group C, GV1001 plus GM-CSF was administered as in group A. Gemcitabine was added at the time of progression and continued at the clinician’s discretion. In group D, gemcitabine alone was given weekly for the first 7 weeks and then in 4-week cycles with three consecutive weekly administrations of gemcitabine followed by a 1-week rest. Vaccine-related adverse events were mild. A telomerase-specific immune response was observed in four/six patients in group A, four/six patients in group B, and two/five patients in group C. An induced ras-specific immune response (antigenic spreading) was observed in 5 of the 17 patients. The cytokine pattern was a Th1-like profile. A treatment-induced telomerase or ras response was also seen in group D. All responses were mild and transient. A significant decrease in regulatory T cells over time was observed in patients in groups A and B (*p* < 0.05). The GV1001 vaccine in combination with chemotherapy appeared to be safe, but the immune response was weak and transient [155]. Of note, a multicenter study (Primovax, NCT00358566) was closed early because of a lack of effects, and measures were taken to augment the magnitude and duration of the immune response to GV1001. Consequently, the GV1001 vaccine was employed in combination with gemcitabine/capecitabine in the TeloVac trial (ISRCTN4382138 and NCT00425360) in PDA patients with locally advanced or metastatic disease. Patients randomized to the control combination chemotherapy (arm 1) received gemcitabine intravenously on days 1, 8, and 15 and capecitabine orally twice daily for 21 days, repeated every 28 days for six cycles. Patients randomized to sequential chemoimmunotherapy (arm 2) received two cycles of combination chemotherapy followed by immunization with an intradermal injection of recombinant GM-CSF followed 10–15 min later by an intradermal injection of GV1001. Immunization was administered on days 1, 3, and 5 in week 1, once in weeks 2, 3, 4, and 6, and then monthly. Patients randomized to concurrent chemoimmunotherapy (arm 3) received combination chemotherapy with GV1001 from day 1 of therapy. The primary vaccination schedule was similarly defined as the first 10 weeks of vaccination, but in this arm, 10 weeks after the start of chemotherapy. The median overall survival times were 7.89, 6.94, and 8.36 months for arms 1, 2, and 3, respectively. The corresponding median times to progression were 6.35, 4.54, and 6.58 months. Delayed type hypersensitivity was positive in 19 (12.3%) of 154 and 47 (20.2%) of 233 patients with sequential and concurrent chemoimmunotherapy, respectively [159]. Nevertheless, cytokine examinations of the TeloVac trial suggested that elevated serum eotaxin levels may predict improved survival in patients who received combined therapy [156,157].

To further investigate the potential antitumor role of VEGFR-2, an oral humanized anti-VEGFR-2 vaccine, namely, VXM01, was tested with success in preclinical mouse models [38,160] and in locally advanced PDA patients [42]. The oral DNA vaccine VXM01 is expected to disrupt the tumor neovasculature and, consequently, inhibit tumor growth. VXM01 potentially combines the advantages of anti-angiogenic therapy and active immunotherapy [42]. In the phase I clinical trial (NCT01486329), 45 patients with locally advanced, unresectable stage IV PDA received four doses of VXM01 on days 1, 3, 5, and 7, starting 3 days after the last dose of gemcitabine administered on days 1, 8, and 15 of a 28-day chemotherapy cycle [42]. The findings of the study provided the rationale for proceeding with a phase II study with the introduction of the blinded placebo patient, as well as a group of patients with a longer life expectancy [42].

Another clinical trial (NCT00622622) targeted VEGFR2-169, an immunogenic peptide derived from VEGFR-2 restricted with HLA-A*24:02, which is the most common HLA-A allele in the Japanese population [36,37,39,40,41,161]. The therapy was administered in PDA patients with unresectable, locally advanced, recurrent, or metastatic disease. Escalating doses of VEGFR2-169 were administered by subcutaneous injection on days 1, 8, 15, and 22 of each 28-day treatment cycle. Gemcitabine was administered intravenously at a fixed dose on days 1, 8, and 15. Repeated cycles of VEGFR2-169 and gemcitabine were administered until patients developed progressive disease or unacceptable toxicity, or for maximum of two cycles, whichever occurred first. The administration of VEGFR2-169 in combination with gemcitabine resulted in the induction of specific cytotoxic T lymphocytes reacting to the VEGFR2-169 peptide in 11 (61%) of the 18 patients, with a disease control rate of 67% [41].

## 9. Conclusions

PDA is associated with an extremely poor survival rate and prognosis when diagnosed at late stages. To date, immunotherapy represents the most promising avenue for improving clinical outcomes for patients with advanced or metastatic PDA by allowing tumor infiltration and modification of the immunosuppressive TME. The development of a therapy that can be offered to all patients with PDA, including those who can undergo surgery and those who are inoperable, is still a significant challenge. The utilization of neoadjuvant therapy and the advancement of diagnostic and, more importantly, prognostic biomarkers that can inform clinicians in selecting the most effective therapies can also make a significant contribution.

As clinical trials progress, it will become increasingly evident which approach is more advantageous. As well as efficacy, factors such as the cost of production will affect the clinical uptake of vaccines. A vaccine by itself may prove inadequate for enabling the immune system to overcome the tumor; therefore, many ongoing trials are combining a vaccine with chemotherapy, radiotherapy, monoclonal antibodies, or ICIs to boost T cell function and activate patients’ antitumor immune responses. 

Developing an efficacious vaccine-based combination therapy presents several challenges in achieving durable responses and overcoming tumor resistance. These include identifying the optimal timing for administering chemotherapy, radiotherapy, monoclonal antibodies, or ICIs and selecting the most appropriate vaccine. In fact, some patients demonstrated a robust antitumor-specific T cell response following vaccine-based immunotherapy, indicating that the treatment effectively stimulated the immune system. However, despite this strong immune activation, there was no clear association with prolonged survival. This suggests that while the immune response was appreciable, it was not sufficient to overcome the aggressive nature of the tumor, highlighting the complexity of translating immune activation into clinical benefit.

However, the efficacy of these clinical trials has varied, with some promising results, but overall, the success of vaccine-based therapies in pancreatic cancer has been challenging because of its immunosuppressive TME and the genetic diversity within patients that complicates the identification of universal and effective tumor antigens. To overcome these challenges, researchers are increasingly focusing on combining vaccines with other therapies, and tailoring vaccines to the specific mutational landscape of an individual’s tumor is a promising strategy. The latter approach is indeed innovative and very promising, but, unfortunately, it is only applicable to the few patients who are candidates for surgery (less than 20% of patients), as it requires a biopsy for sequencing and identification of the mutations that give rise to neoantigens. This excludes the majority of patients who have an advanced or metastatic tumor at diagnosis. Therefore, further efforts are urgently needed to identify tumor antigens that are universally expressed in all patients, in parallel with the identification of biomarkers that predict which patients are most likely to respond to vaccine-based therapies.
